# Perimyocarditis as First Manifestation of Systemic Lupus Erythematosus Successfully Treated with Heart Failure and Immunosuppressive Therapy

**DOI:** 10.3390/jcdd10040134

**Published:** 2023-03-23

**Authors:** Marina Ikić Matijašević, Petra Grubić Rotkvić, Zrinka Planinić, Lucija Ikić, Ines Zadro Kordić, Edvard Galić

**Affiliations:** 1 Internal Medicine Clinic, University Hospital Sveti Duh, 10 000 Zagreb, Croatia; petra.grubic84@gmail.com (P.G.R.); edvard.galic1@gmail.com (E.G.); 2 School of Medicine, University of Zagreb, 10 000 Zagreb, Croatia; 3 Department of Anatomy and Physiology, University of Applied Health Sciences, 10 000 Zagreb, Croatia

**Keywords:** systemic lupus erythematosus, heart failure, myocarditis, pericarditis, speckle tracking echocardiography, transthoracic echocardiography, immunosuppressants, glucocorticoids, therapy, biomarkers

## Abstract

Systemic lupus erythematosus (SLE) myocarditis is presumed to be rare, but associated with adverse outcomes. If SLE diagnosis has not previously been established, its clinical presentation is often unspecific and difficult to recognize. Furthermore, there is a lack of data in the scientific literature regarding myocarditis and its treatment in systemic immune-mediated diseases, leading to its late recognition and undertreatment. We present the case of a young woman whose first lupus manifestations included acute perimyocarditis, among other symptoms and signs that provided clues to the diagnosis of SLE. Transthoracic and speckle tracking echocardiography were helpful in detecting early abnormalities in the myocardial wall thickness and contractility while waiting for cardiac magnetic resonance. Since the patient presented with acute decompensated heart failure (HF), HF treatment was promptly started in parallel with immunosuppressive therapy, with a good response. In the treatment of myocarditis with heart failure, we were guided by the clinical signs, echocardiographic findings, biomarkers of myocardial stress, necrosis, and systemic inflammation, as well as markers of SLE disease activity.

## 1. Introduction

Systemic lupus erythematosus (SLE) is an autoimmune disease more common in women of childbearing age and can affect any organ. Cardiac disease is frequent among SLE patients, and can manifest as conduction abnormalities, valvular, pericardial, myocardial, endocardial, and coronary artery disease, with cardiovascular events being the major cause of premature death [1]. Despite a general decline in SLE mortality over the past decades, cardiovascular mortality remains unchanged [2]. Pericarditis is the most common echocardiographic finding, occurring in more than half of SLE patients [3]. Lupus myocarditis has a prevalence of 0.4–16%, and since it can be life-threatening, we must suspect myocarditis in every SLE patient with HF [4]. Cardiac magnetic resonance (CMR) appears to be the most precise non-invasive diagnostic tool, although not widely available, while right heart catheterization with endomyocardial biopsy (EMB) should be considered when it is not entirely clear that SLE is the cause of myocarditis. Transthoracic echocardiography (TTE) may reveal abnormalities of the left ventricle (LV) systolic and diastolic function, and both global and segmental areas of hypokinesis can be detected. Depending on the degree of myocardial inflammation, chamber size and myocardial wall thickness reflecting interstitial edema can vary [5]. In addition, speckle tracking echocardiography (STE) can detect subclinical abnormalities in myocardial contractility, and could be helpful in diagnosing myocarditis, especially in its initial stages [6]. Moreover, it was shown that the localizations of myocarditis area that showed in CMR correspond to areas with depressed longitudinal strain in echocardiography [6]. Acute myocarditis in lupus is often accompanied by pericarditis, and treatment of both has not been assessed in controlled trials and is so far mostly based on expert opinions and case reports published in the literature. Here, we present a patient in whom acute heart failure (HF) due to acute perimyocarditis was the first SLE manifestation and who was successfully treated with high doses of methylprednisolone (MP) and hydroxychloroquine (HCQ) in parallel with standard HF therapy.

## 2. Case Description

A 30-year-old woman was hospitalized at the internal medicine department due to recurrent biliary colic and suspicion of the development of acute calculous cholecystitis with a mildly elevated erythrocyte sedimentation rate (ESR) and C-reactive protein (CRP). Treatment with analgesics, spasmolytics, and antibiotics was started. Despite the treatment, three days later, the patient became febrile (>38.5 °C), developed a maculopapular rash on the arms and torso, a malar rash, and had dyspnea with elevated N-terminal pro b-type natriuretic peptide (NT-proBNP) (9373 ng/L, normal range: <208 ng/L) and without other clinical signs of heart failure. Additionally, our patient reported chest pain with elevated hs-troponin I (293 ng/L, normal range: <39 ng/L), with sinus tachycardia in the ECG but without signs of ischemia in the serially repeated ECGs (Table 1). The patient also had a low level of albumin, an elevated level of fibrinogen, and a normal value of ferritin, as markers of acute inflammation.

**Figure 1:**
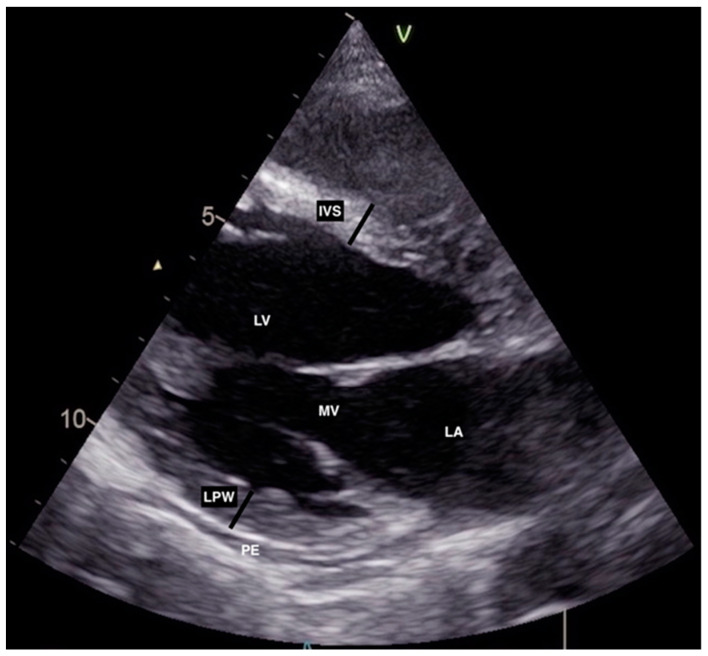
Initial transthoracic echocardiography showing left ventricular wall thickness (IVS, LPW) within normal range in parasternal long axis (PLAX) view. IVS: interventricular septum, LA: left atrium, LV: left ventricle, LPW: left posterior wall, MV: mitral valve, PE: pericardial effusion.

**Figure 2:**
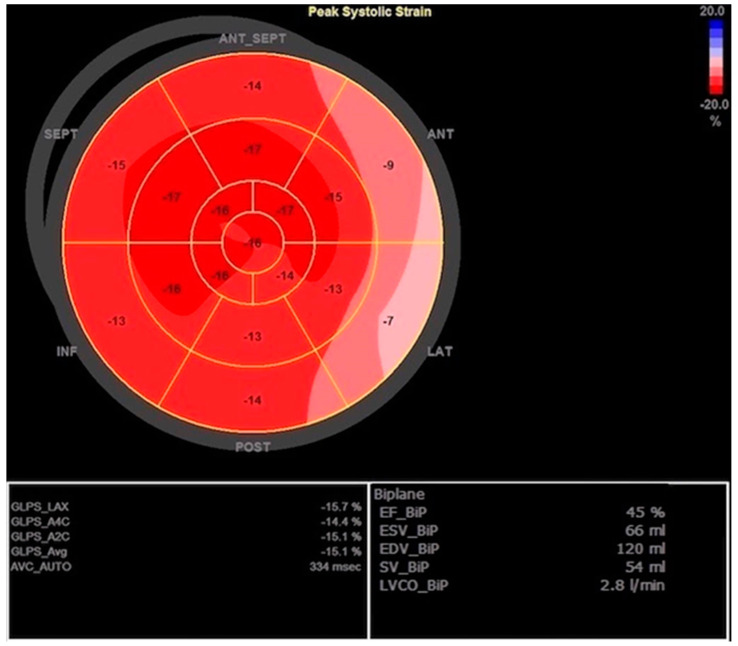
Initial transthoracic echocardiography with mildly reduced left ventricular ejection fraction (EF) calculated using Simpson biplane method and reduced global longitudinal peak strain (GLPS) with reduced values in basal segment of the anterior, septal, and lateral walls.

Furthermore, abdominal ultrasound at that time showed no signs of acute cholecystitis, while on TTE, there was hypo-contractility in the basal segment of the anterior, septal, and lateral walls with preserved wall thickness and LV diameter, mildly reduced LV ejection fraction (EF) (45%), reduced global longitudinal peak strain (GLPS) -15.1%, moderate mitral regurgitation (MR), diastolic dysfunction (DD) grade 3, and a small pericardial effusion (2–3 mm) (Figure 1 and Figure 2).

**Figure 3:**
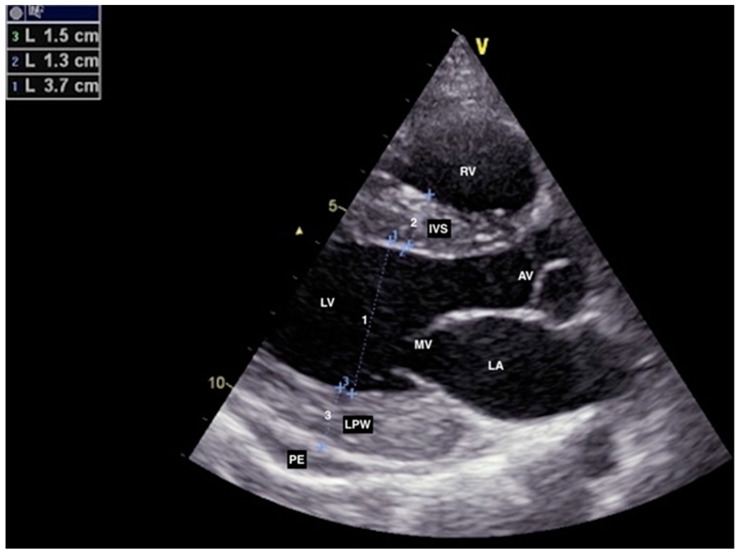
Second transthoracic echocardiography showing markedly increased thickness of left ventricle (LV) walls (IVS, LPW) in parasternal long axis (PLAX) view. AV: aortic valve, IVS: interventricular septum, LA: left atrium, LPW: left posterior wall, MV: mitral valve, PE: pericardial effusion, RV: right ventricle.

**Figure 4:**
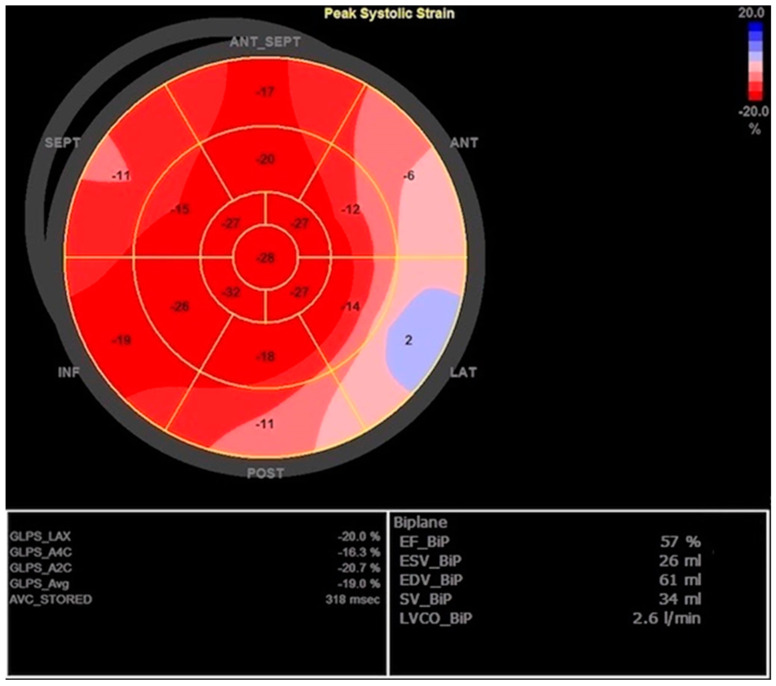
Second transthoracic echocardiography with improved left ventricle ejection fraction (EF) and improved global longitudinal peak strain (GLPS) but still noticeable reduced regional strain values.

Since there was concern about a possible associated allergic reaction (rash) during the treatment with antibiotics, the patient immediately received a low dose of corticosteroids and antihistamines while extensive evaluation was initiated in parallel with guideline-directed treatment for HF. Blood and urine cultures were negative, as were viral panels for cardiotropic infectious agents. All tumor markers and the interferon-gamma release assay for latent tuberculosis infection were in the reference range. A clinical immunologist examined the patient and recommended diagnostic workup under suspicion of SLE. Coronary angiography did not show obstructive coronary artery disease. Since the patient manifested symptomatic HF with a slightly reduced EF but with the possibility of fast deterioration, we introduced diuretics, beta-blockers, aldosterone antagonists, and sacubitril/valsartan, as well as ibuprofen due to signs of pericarditis. Interestingly, a few days later, the TTE showed a markedly increased thickness of the LV walls with normal EF (57%), GLPS of −19%, mild MR, DD grade 1, and progression of pericardial effusion to 10 mm. The GLPS abnormalities were again found in the basal segments of the anterior, septal, and lateral walls (Figure 3 and Figure 4).

**Figure 5:**
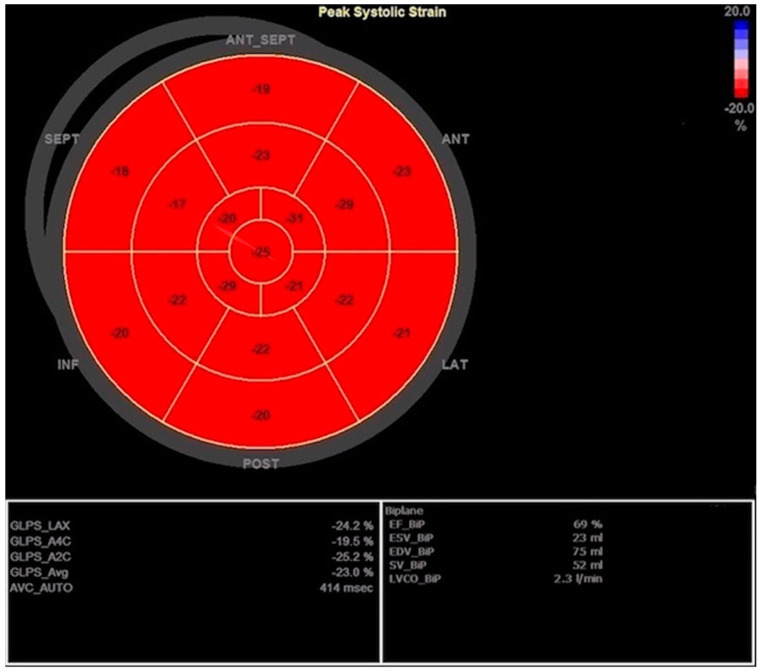
Completely recovered left ventricle ejection fraction (EF) and global longitudinal peak strain (GLPS) with no signs of hypokinesis 3 weeks after initiating guideline-directed heart failure therapy concomitantly with immunosuppressive therapy.

This increased thickness of myocardial walls in the second TTE likely reflected interstitial edema due to suspected inflammation, and the rapid EF improvement was explained by better loading conditions due to diuretics and HF therapy. Nevertheless, depressed GLPS was still noted within several segments of the LV, raising suspicion of myocarditis. After immunological workup, the diagnosis of SLE was made; the 2019 EULAR/ACR criteria for SLE were fulfilled: leukopenia presented on several occasions before according to available medical documentation, strongly positive antinuclear antibodies, complement (C) consumption low C3, pericarditis, myocarditis, fever, and acute cutaneous lupus (Table 1). Additionally, the patient reported photosensitivity and recurrent cervical lymphadenopathy, and had persistently low lymphocyte count with occasional thrombocytopenia, but not less than 100 × 10^9^/L. Therefore, in addition to HF therapy, HCQ 200 mg and MP 2 mg/kg/day were introduced, and ibuprofen was ceased. Soon after initiating the above-mentioned therapy, the patient reported regression of all symptoms, and there was a significant decrease in NT-proBNP, normalization of leukocyte and platelet count, troponin, ESR, CRP, and fibrinogen, and the MP was reduced to 0.5 mg/kg/day. Meanwhile, we obtained CMR that confirmed the myocarditis diagnosis, and the results were comparable with abnormalities detected in the TTE. More precisely, cardiac magnetic resonance performed 2 weeks after the first heart failure manifestation described focal tissue edema, especially in the anterior region and the interventricular septum, in the area that was most thickened in the TTE. After only 3 weeks of therapy, TTE was completely normal (Figure 5) with an EF of 69%, and we tapered MP to 0.3 mg/kg/day.

Sacubitril/valsartan and aldosterone antagonists were completely discontinued after 6 months, and the dose of MP was gradually decreased with total discontinuation after 12 months (after 6 months, MP dose was 8 mg daily). Now, 24 months after the first manifestation of SLE, the patient is still in complete clinical and immunological disease remission and is continuously taking bisoprolol at a minimal dosage and 200 mg of HCQ (Table 1).

## 3. Discussion

Clinicians must be aware of unusual SLE presentations such as acute HF due to SLE-myocarditis, with only 0.37% of patients presenting as such, but with potentially fatal outcomes: cardiogenic shock or malignant arrhythmias [7]. The diagnosis of SLE itself, especially SLE-myocarditis, is challenging in clinical practice if the patient has not had a previous diagnosis of lupus or lupus signs and symptoms. Although EMB is considered the gold standard for the diagnosis of myocarditis, it is not a routine procedure because it is invasive and has a risk of sampling error. Consequently, to establish the correct diagnosis of SLE-myocarditis, we must combine the patient’s previous medical history, current signs and symptoms, laboratory tests, TTE with STE, CMR, and EMB if available, and exclude other common causes of myocarditis. In our case, we established a diagnosis of SLE according to the 2019 EULAR/ACR classification criteria and after the exclusion of other causes of myocarditis. In the treatment of myocarditis in SLE, we were guided by the 2016 ESC guidelines for HF valid at that time, the position statement of the ESC Working Group on Myocardial and Pericardial Disease, EULAR recommendations for the management of SLE, previously reported case reports, expert opinions, and reviews [8,9,10,11,12,13,14,15]. Most of the treatment evidence is derived from case reports, case–control, and cohort studies. According to the literature, the treatment of lupus myocarditis requires an initial period of intensive immunosuppression to decrease disease activity, followed by a longer period of less intensive therapy to consolidate the response and prevent relapses [9,12]. The standard of care for patients with severe myocarditis includes high-dose glucocorticoids plus intravenous cyclophosphamide or another immunosuppressant such as rituximab, and azathioprine is usually used as steroid-sparing maintenance therapy [9,10,12,15]. There are few data in the literature regarding the optimal immunosuppressive regimen in the treatment of SLE myocarditis with mildly reduced ejection fraction, and some data suggest that patients with less severe disease exhibited good left ventricular ejection fraction recovery without cyclophosphamide or other immunosuppressants [15,16]. Our patient was initially treated with a high dose of MP: 2 mg/kg/day (with the possible implication of early-started low-dose corticosteroids due to rash and concern of an allergic reaction), and the high dose was reduced to a moderate dose (0.3 mg/kg/day) after TTE and laboratory confirmation of myocardial recovery with further gradual dose reduction. Ibuprofen was stopped since non-steroidal anti-inflammatory drugs (NSAIDs) could enhance inflammation and increase mortality, which has been shown in animal models of myocarditis, although recent findings suggest that treatment with NSAIDs does not affect outcome in patients with acute myocarditis or myopericarditis [10,11,17]. In our opinion, patients with HF due to SLE should be treated in parallel with HF guideline-directed therapy to reduce the risk of further myocardial damage and potential complications, although the optimal duration of the treatment is not yet well established. Additionally, the optimal duration of glucocorticoid therapy is not known in lupus myocarditis, but we were guided by the patient’s clinical signs and symptoms and laboratory and TTE findings. The treatment should be started as soon as possible to prevent further damage by suppressing the inflammation that leads to heart remodeling and fibrosis. Furthermore, in relation to changes detected in TTE with STE, a combination of widely available laboratory parameters/biomarkers such as troponin and NT-proBNP (biomarkers of myocardial injury), anti-dsDNA, C3 and C4 (biomarkers of SLE disease activity), and ESR, CRP, albumin, and fibrinogen (biomarkers of systemic inflammation) could be used not only for early diagnosis but also for SLE-myocarditis follow-up and therapy management [18,19]. In the absence of a rapid response to glucocorticoids, another immunosuppressant such as cyclophosphamide, mycophenolate mofetil, azathioprine, or rituximab should be introduced into the therapy [14,15,20]. Additionally, we must bear in mind that the treatment of myocarditis in systemic autoimmune or autoinflammatory disease should be tailored to each individual patient depending on which organs are simultaneously affected by the disease. We believe that timely initiated immunosuppressive glucocorticoid therapy in parallel with HF therapy with neurohumoral blockade was the key to successful treatment in the case of our patient.

## 4. Conclusions

Despite its low prevalence, lupus myocarditis should be considered as a differential diagnosis in patients with new-onset heart failure. Early diagnosis, treatment with immunosuppressants according to the severity of myocarditis, and heart failure therapy, as well as close monitoring of the response to therapy, may lead to a favorable outcome. This case demonstrates a rare presentation of SLE myocarditis with rapid clinical recovery on glucocorticoid and heart failure therapy. Of course, an optimal treatment strategy cannot be made on the basis of a single case report. Randomized trials with larger numbers of patients conducted by cardiologists and clinical immunologists/rheumatologists are necessary to provide guidelines and recommendations for diagnosing and managing myocarditis in autoimmune and autoinflammatory systemic diseases.

## Figures and Tables

**Table 1:** Laboratory data upon admission and 24 months after initiation of heart failure and immunosuppressive therapy.

	Reference Range	Admission	After 24 Months
White blood cell count (10^9^/L)	3.4–9.7	3.1	6.9
Hemoglobin (g/L)	138–175	116	137
Platelets (10^9^/L)	158–424	132	193
CRP (mg/dL)	<5.0	26.5	3.5
ESR (mm/3.6 ks)	4–24	28	11
Albumin (g/L)	41–51	36	44
Ferritin (µg/L)	10–158	61	12
Fibrinogen (g/L)	1.8–3.5	3.9	2.2
Procalcitonin (µg/L)	0.5–2	1.30	<0.5
Creatinine (µmol/L)	49–90	68	51
24 h urine protein (mg/24 h)	<150	54	145
hs-troponin I (ng/L)	<39	293	36
NT-proBNP (ng/L)	<208.4	9373	135
Direct antiglobulin test		Negative	
C3 (g/L)	0.89–1.87	0.72	1.0
C4 (g/L)	0.17–0.38	0.17	0.28
Antinuclear antibody *	Positive > 1	>55	Not performed
Anti-dsDNA antibody * (IU/mL)	Positive > 15	<0.7	1.2
Anti-Smith (Sm) antibody		Not performed	Not performed
Anticardiolipin antibody IgM * (MPL-U/mL)	Positive > 40	1.1	0.7
Anticardiolipin antibody IgG * (GPL-U/mL)	Positive > 40	1.2	0.9
Beta2-Glycoprotein I IgM * (EliA U/mL)	Positive > 10	<2.4	<2.4
Beta2-Glycoprotein I IgG * (EliA U/mL)	Positive > 10	<0.8	<0.8
Lupus anticoagulant		Negative	Not performed

CRP: C-reactive protein, ESR: erythrocyte sedimentation rate, NT-proBNP: N-terminal pro b-type natriuretic peptide, C: complement, * FEIA Phadia 200.

## Data Availability

The original data generated and analyzed for this study are included in the published article. Further inquiries can be directed to the corresponding author.

## References

[1] Tincani A., Rebaioli C.B., Taglietti M., Shoenfeld Y. (2006). Heart involvement in systemic lupus erythematosus, anti-phospholipid syndrome and neonatal lupus. Rheumatology.

[2] Björnådal L., Yin L., Granath F., Klareskog L., Ekbom A. (2004). Cardiovascular disease a hazard despite improved prognosis in patients with systemic lupus erythematosus: Results from a Swedish population based study 1964–1995. J. Rheumatol..

[3] Dein E., Douglas H., Petri M., Law G., Timlin H. (2019). Pericarditis in lupus. Cureus.

[4] Tani C., Elefante E., Arnaud L., Barreira S.C., Bulina I., Cavagna L., Costedoat-Chalumeau N., Doria A., Fonseca J.E., Franceschini F. (2022). Rare clinical manifestations in systemic lupus erythematosus: A review on frequency and clinical presentation. Clin. Exp. Rheumatol..

[5] Felker G.M., Boehmer J.P., Hruban R.H., Hutchins G.M., Kasper E.K., Baughman K.L., Hare J.M. (2000). Echocardiographic findings in fulminant and acute myocarditis. J. Am. Coll. Cardiol..

[6] Uziębło-Życzkowska B., Mielniczuk M., Ryczek R., Krzesiński P. (2020). Myocarditis successfully diagnosed and controlled with speckle tracking echocardiography. Cardiovasc. Ultrasound.

[7] Tanwani J., Tselios K., Gladman D.D., Su J., Urowitz M.B. (2018). Lupus myocarditis: A single center experience and a comparative analysis of observational cohort studies. Lupus.

[8] Ponikowski P., Voors A.A., Anker S.D., Bueno H., Cleland J.G., Coats A.J., Falk V., González-Juanatey J.R., Harjola V.P., Jankowska E.A. (2016). 2016 ESC Guidelines for the diagnosis and treatment of acute and chronic heart failure: The Task Force for the diagnosis and treatment of acute and chronic heart failure of the European Society of Cardiology (ESC). Developed with the special contribution of the Heart Failure Association (HFA) of the ESC. Eur. J. Heart Fail..

[9] Caforio A.L.P., Adler Y., Agostini C., Allanore Y., Anastasakis A., Arad M., Böhm M., Charron P., Elliott P.M., Eriksson U. (2017). Diagnosis and management of myocardial involvement in systemic immune-mediated diseases: A position statement of the European Society of Cardiology Working Group on Myocardial and Pericardial Disease. Eur. Heart J..

[10] Caforio A.L., Pankuweit S., Arbustini E., Basso C., Gimeno-Blanes J., Felix S.B., Fu M., Heliö T., Heymans S., Jahns R. (2013). Current state of knowledge on aetiology, diagnosis, management, and therapy of myocarditis: A position statement of the European Society of Cardiology Working Group on Myocardial and Pericardial Diseases. Eur. Heart J..

[11] Adler Y., Charron P., Imazio M., Badano L., Barón-Esquivias G., Bogaert J., Brucato A., Gueret P., Klingel K., Lionis C. (2015). 2015 ESC Guidelines for the diagnosis and management of pericardial diseases: The task force for the diagnosis and management of pericardial diseases of the European Society of Cardiology (ESC)endorsed by: The European Association for Cardio-Thoracic Surgery (EACTS). Eur. Heart J..

[12] Fanouriakis A., Kostopoulou M., Alunno A., Aringer M., Bajema I., Boletis J.N., Cervera R., Doria A., Gordon C., Govoni M. (2019). 2019 update of the EULAR recommendations for the management of systemic lupus erythematosus. Ann. Rheum. Dis..

[13] Durrance R.J., Movahedian M., Haile W., Teller K., Pinsker R. (2019). Systemic lupus erythematosus presenting as myopericarditis with acute heart failure: A case report and literature review. Case Rep. Rheumatol..

[14] Zhang L., Zhu Y.L., Li M.T., Gao N., You X., Wu Q.J., Su J.M., Shen M., Zhao L.D., Liu J.J. (2015). Lupus myocarditis: A case-control study from China. Chin. Med. J..

[15] Xibillé-Friedmann D., Pérez-Rodríguez M., Carrillo-Vázquez S., Álvarez-Hernández E., Aceves F.J., Ocampo-Torres M.C., García-García C., García-Figueroa J.L., Merayo-Chalico J., Barrera-Vargas A. (2019). Clinical practice guidelines for the treatment of systemic lupus erythematosus by the Mexican College of Rheumatology. Guía de práctica clínica para el manejo del lupus eritematoso sistémico propuesta por el Colegio Mexicano de Reumatología. Reumatol. Clin..

[16] Thomas G., Cohen Aubart F., Chiche L., Haroche J., Hié M., Hervier B., Costedoat-Chalumeau N., Mazodier K., Ebbo M., Cluzel P. (2017). Lupus myocarditis: Initial presentation and longterm outcomes in a multicentric series of 29 patients. J. Rheumatol..

[17] Mirna M., Schmutzler L., Topf A., Boxhammer E., Sipos B., Hoppe U.C., Lichtenauer M. (2022). Treatment with non-steroidal anti-inflammatory drugs (NSAIDs) does not affect outcome in patients with acute myocarditis or myopericarditis. J. Cardiovasc. Dev. Dis..

[18] Gulhar R., Ashraf M.A., Jialal I. (2019). Physiology, Acute Phase Reactants.

[19] Suresh A., Martens P., Tang W.H.W. (2022). Biomarkers for myocarditis and inflammatory cardiomyopathy. Curr. Heart Fail. Rep..

[20] Zagelbaum Ward N.K., Linares-Koloffon C., Posligua A., Gandrabur L., Kim W.Y., Sperber K., Wasserman A., Ash J. (2022). Cardiac manifestations of systemic lupus erythematous: An overview of the incidence, risk factors, diagnostic criteria, pathophysiology and treatment options. Cardiol. Rev..

